# Prognostic Value of Postoperative Neutrophil and Albumin: Reassessment One Month After Gastric Cancer Surgery

**DOI:** 10.3389/fonc.2021.633924

**Published:** 2021-03-23

**Authors:** Ali Guner, Minah Cho, Yoo-Min Kim, Jae-Ho Cheong, Woo Jin Hyung, Hyoung-Il Kim

**Affiliations:** ^1^ Department of Surgery, Yonsei University College of Medicine, Seoul, South Korea; ^2^ Faculty of Medicine, Department of General Surgery, Karadeniz Technical University, Trabzon, Turkey; ^3^ Gastric Cancer Center, Yonsei Cancer Center, Seoul, South Korea; ^4^ Open NBI Convergence Technology Research Laboratory, Severance Hospital, Yonsei Cancer Center, Yonsei University College of Medicine, Seoul, South Korea

**Keywords:** stomach neoplasms, inflammation, neutrophils, albumin, prognosis, survival

## Abstract

**Objective:**

The prognostic value of postoperative parameters reflecting the inflammatory and nutritional status of patients undergoing cancer surgery has been rarely studied. This study investigated the prognostic value of inflammatory and nutritional parameters measured preoperatively and 1 month after curative gastrectomy for gastric cancer.

**Methods:**

Data from a prospectively maintained database of 1,194 patients with gastric cancer who underwent curative surgery in 2009–2018 were retrospectively reviewed. Demographics, clinicopathologic characteristics, operative data, survival data, and laboratory parameters were extracted. Neutrophil counts, lymphocyte counts, and albumin levels before surgery and 1 month postoperatively were analyzed.

**Results:**

In multivariable analysis adjusted for age, sex, and pathologic stage, high neutrophil count (hazard ratio [HR] 1.09, 95% confidence interval [CI] 1.01–1.17, p = 0.022) and low albumin (HR 0.45, 95% CI 0.27–0.74, p = 0.002) 1 month postoperatively were independent prognostic factors for overall survival. High neutrophil count (HR 1.09, 95% CI 1.02–1.16, p = 0.015) 1 month postoperatively was also an independent prognostic factor for recurrence-free survival after adjusting for age, sex, body mass index, extent of gastrectomy, and pathologic stage. Patients were classified into risk groups based on thresholds of 4.2 × 10^3^ cells/mm^3^ and 4.1 g/dl for 1-month neutrophil count and albumin. High-risk groups had a significantly worse prognosis than low-risk groups for overall survival (HR 5.87, 95% CI 3.28–10.51, p <0.001) and recurrence-free survival (HR 1.52, 95% CI 1.07–2.16, p = 0.021).

**Conclusions:**

Neutrophil count and albumin level 1 month after curative surgery reflect long-term prognosis better than preoperative values. These parameters can be used to stratify patients with the same stage into different prognostic groups.

Gastric cancer is one of the common cancer types worldwide. Although surgical treatment is known as the only curative treatment option, a better survival has been achieved with developing neoadjuvant and adjuvant treatment modalities. Treatment decision-making for patients with gastric cancer is determined primarily by TNM staging. Increasing evidence has shown that inflammatory and nutritional status also influence long-term outcomes. However, assessing this status is usually conducted in the preoperative period. It is hypothesized that laboratory values obtained after curative treatment may reflect the prognosis better than the preoperative values. In the present study, we evaluated the associations between laboratory values obtained 1 month after curative surgery and survival. Our results showed that 1-month neutrophil and albumin values were independent prognostic factor of overall survival and 1-month neutrophil count was an independent prognostic factor of recurrence-free survival. Risk stratification according to these laboratory parameters revealed worse overall and recurrence-free survival in high-risk groups than in low-risk groups.

## Introduction

Gastric cancer is one of the most common solid organ malignancies worldwide, and radical surgery remains the only potentially curative treatment. Risk stratification based on prognostic factors is essential for predicting long-term outcomes and determining the need for adjuvant therapy (1).

TNM staging is the standard method of prognosis prediction; however, parameters reflecting inflammatory status may also have a role in prognosis prediction. Preoperative neutrophil count, neutrophil-to-lymphocyte ratio (NLR), platelet-to-lymphocyte ratio (PLR), systemic immune-inflammation index (SII), prognostic nutritional index (PNI), and albumin level have all been associated with survival (2–7). We previously demonstrated that among many complex parameters, the simple parameter of a high preoperative neutrophil count is a useful prognostic marker for poor overall survival (OS) and recurrence-free survival (RFS) (7, 8).

While a number of gastric cancer studies focused on preoperative status, few studies explored the implications of postoperative parameters (3–5). Patients are often reassessed 1 month after gastrectomy, prior to initiating adjuvant therapy. It is assumed that nutritional defects and inflammation associated with the disease and surgery would have been resolved by this time.

We hypothesized that parameters obtained 1 month after curative surgery may reflect patient status and estimate long-term outcomes. To explore this hypothesis, we investigated the prognostic value of inflammation and nutritional parameters measured preoperatively and 1 month after curative gastrectomy for gastric cancer.

## Materials and Methods

From a prospectively maintained database, we retrospectively reviewed the data of 1,932 consecutive patients with histologically confirmed gastric adenocarcinoma who underwent radical gastrectomy at the Severance Hospital of Yonsei University Health System (Seoul, Korea) between March 2009 and June 2018. The exclusion criteria for the study were as follows: (i) non-curative surgery; (ii) metastatic disease; (iii) malignancy of another organ; (iv) neoadjuvant treatment; (v) emergency surgery; (vi) active infection or immunologic disease; (vii) major postoperative complication (Clavien-Dindo classification Grade 3 or higher); or (viii) incomplete laboratory test data. This study was approved by the Institutional Review Board of Severance Hospital (4-2020-1084).

Clinicopathologic characteristics, including age, sex, body mass index (BMI), medical comorbidities, American Society of Anesthesiologists physical status score, pathologic stage, and adjuvant treatment, were extracted from the database. Perioperative data, including surgical approach, extent of lymphadenectomy and gastrectomy, operation time, intraoperative blood loss, and combined resection, were likewise extracted. The extent of lymphadenectomy and gastrectomy was performed in accordance with Korean and Japanese guidelines (9, 10). The pathologic stage was determined according to the 8th edition of the American Joint Committee on Cancer staging system (11). Adjuvant chemotherapy was recommended for patients with stage II or higher disease.

Patients were followed according to a fixed schedule: 1 month after gastrectomy, every 3 months for 2 years, and then every 6 months for 3 years thereafter. The follow-up schedule consisted of clinical and laboratory examinations, imaging, and endoscopic evaluation. The last database update was in April 2020. Neutrophil counts, lymphocyte counts, and albumin levels were obtained from the results of blood samples collected within 1 week before surgery (preop); on the day of surgery (D0); on postoperative days 1 (D1), 3 (D3), and 5 (D5); and 1 month after surgery (M1). Neutrophil, lymphocyte, and albumin levels at one month after surgery was presented as neutrophil-M1, lymphocyte -M1, and albumin -M1 respectively.

The study endpoints were OS and RFS. Survival times were calculated from the date of surgery to the time of death for OS and clinical, histologic, or radiologic recurrence for RFS. Data from the remaining patients were censored at the last follow-up evaluation. Deaths caused by cancer progression or cancer-related long-term complications were defined as cancer-related deaths, while deaths unrelated to the malignancy were defined as non-cancer deaths. Recurrence pattern was defined as peritoneum-associated or extra-peritoneal recurrence. Any recurrence involving the peritoneum (peritoneum-only or combined) was defined as a peritoneum-associated recurrence. Any recurrence not involving the peritoneum (locoregional recurrence including the anastomosis site or lymph nodes, distant recurrence including hematogenous metastasis, and metastasis at extra-abdominal lymph nodes) was defined as an extra-peritoneal recurrence.

### Statistical Analysis

Statistical analysis was performed using R software (R Foundation for Statistical Computing, Vienna, Austria) with the required R packages. Data were presented as mean ± standard deviation or median (1st–3rd quartiles) for continuous variables, and as frequencies (percentages) for categorical variables. The normality distribution of continuous variables was assessed with the Shapiro–Wilk test and visual inspection of normal Q–Q plots. Pearson correlation test was used for the correlation between variables.

To evaluate the prognostic effects of laboratory values on survival, we first analyzed clinicopathologic factors influencing long-term prognosis. Cox proportional hazards regression models were used, and the purposeful selection method described by Hosmer and Lemeshow was applied to the factors (12). Briefly, any variable with a p-value <0.25 on univariable testing and all variables of known clinical importance were entered into the multivariable model. Coefficient changes and interactions were also assessed, and variables with a p-value <0.05 were considered for the final model. The selected clinicopathologic factors were used to test the prognostic effects of laboratory values in further multivariable analysis. Schoenfeld residuals and the Grambsch–Therneau test were used to test the proportional hazards assumption. Martingale residuals and penalized spline fit were used to evaluate the linearity assumption. All continuous variables were assessed for definitive evidence of non-linear relationships using penalized smoothing splines (pspline function from the R Survival Package), and linear variables were used in the Cox regression models without grouping by cut-off value. Univariable (only laboratory values) and multivariable analysis with backward selection based on the Akaike’s information criterion (clinicopathologic factors and all laboratory values) were used in the regression models. The results were described as hazard ratios (HRs) with 95% confidence intervals (CIs).

Survival outcomes were analyzed using Kaplan–Meier curve analysis, and comparisons between groups were performed using the log-rank test. Various cut-offs were analyzed, and thresholds were selected based on the highest log-rank score and lowest p-value. P-values were two-sided and statistical significance was defined as a p-value <0.05 unless otherwise stated.

## Results

Of the 1,194 patients with gastric cancer enrolled in the study, 751 (62.9%) were male and 443 (37.1%) were female (**Table 1**). The median age was 60 (51–68) years. There were 801 (67.1%) patients with stage I cancer, 171 (14.3%) patients with stage II cancer, and 222 (18.6%) patients with stage III cancer. A total of 355 (29.7%) patients received adjuvant chemotherapy. The median follow-up time for patients with no event was 39 (26–58) months. Recurrence was detected in 127 (10.6%) patients, and 101 (8.5%) patients died. In patients with recurrence, 74 (58%) had peritoneum-associated recurrence, while 53 (42%) experienced extra-peritoneal recurrence. Among patients who died during the follow-up period, 85 (84%) died from gastric cancer-related causes, while 16 (16%) died from non-cancer causes.

**Table 1:** Clinicopathologic characteristics of all included patients.

Variable		n (%) or median (Q1–Q3) (N = 1194)
Age (y)		60 (51–68)
Sex	Male	751 (62.9)
	Female	443 (37.1)
Body mass index (kg/m^2^)		23.6 (21.5–25.9)
ASA physical status score	I	308 (25.8)
	II	625 (52.3)
	III	239 (20.0)
	IV	22 (1.8)
Comorbidity	No	542 (45.4)
	Yes	652 (54.6)
Previous abdominal surgery	No	722 (60.5)
	Yes	472 (39.5)
CEA (ng/ml)		1.8 (1.1–2.7)
CA 19-9 (U/ml)		7.9 (4.5–13.7)
Neutrophil count (× 10^3^ cell/mm^3^)		3.6 (2.8–4.5)
Lymphocyte count (× 10^3^ cell/mm^3^)		2.0 (1.6–2.4)
Albumin (g/dl)		4.4 (4.2–4.7)
NLR		1.8 (1.3–2.4)
PNI		54.9 (51.2–58.3)
Approach	Open	212 (17.8)
	Laparoscopy	558 (46.7)
	Robotic	424 (35.5)
Extent of lymphadenectomy	D1+	752 (63.0)
	D2	442 (37.0)
Extent of gastrectomy	Subtotal	962 (80.6)
	Total	232 (19.4)
Operation time (min)		160.0 (130.0–200.0)
Intraoperative blood loss (ml)		41.0 (20.0–90.0)
Combined resection	No	1057 (88.5)
	Yes	137 (11.5)
Transfusion	No	1169 (97.9)
	Yes	25 (2.1)
T classification	T1	757 (63.4)
	T2	122 (10.2)
	T3	146 (12.2)
	T4	169 (14.2)
N classification	N0	821 (68.8)
	N1	138 (11.6)
	N2	101 (8.5)
	N3	134 (11.2)
Pathologic stage	I	801 (67.1)
	II	171 (14.3)
	III	222 (18.6)

ASA, American Society of Anesthesiologists; CEA, carcinoembryonic antigen; NLR, neutrophil/lymphocyte ratio; PNI, Prognostic Nutritional index; Q1–Q3, first quartile–third quartile.

**Figure 1:**
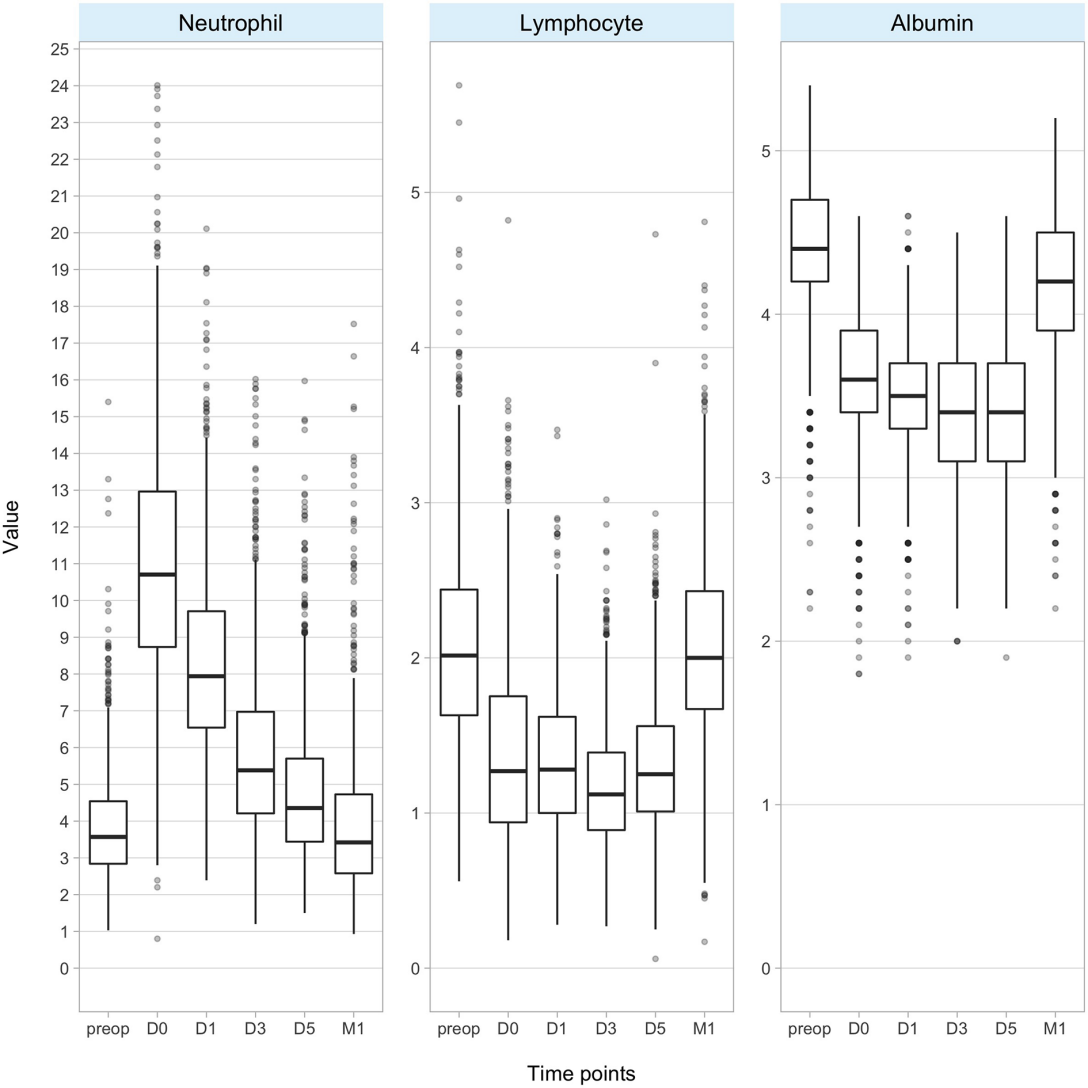
Box plots of neutrophil count, lymphocyte count, and albumin level at different time points. Y-axis represents × 10^3^ cells/ml for neutrophils and lymphocytes and g/dl for albumin. (D, day; M, month; preop, preoperative).

Postoperative changes in laboratory values are presented in **Figure 1**. Neutrophil counts increased on D0, then gradually decreased and reached a median value of 3.3 × 10^3^ cell/mm^3^ (2.5–4.5) at 1 month after surgery. Lymphocyte counts and albumin levels decreased initially after surgery and then returned to median values of 2 × 10^3^ cell/mm^3^ (1.7–2.4) and 4.2 g/dl (4.0–4.5), respectively, at 1 month after surgery. The correlation matrix and the correlation coefficients between the variables were presented in **Supplemental Figure 1**.

### Univariable and Multivariable Analyses for Overall Survival and Recurrence-Free Survival

In Cox regression analysis of clinicopathologic variables, age, sex, and pathologic stage were identified as independent prognostic factors for OS, and age, sex, BMI, extent of gastrectomy, and pathologic stage were identified as independent prognostic factors for RFS. These factors were adjusted for during multivariable analysis of laboratory variables (pre- and postoperative neutrophil counts, lymphocyte counts, and albumin levels). Most of these laboratory values were statistically significant prognostic factors in univariable analysis (**Table 2**). In adjusted multivariable analysis, both neutrophil-M1 (HR 1.09, 95% CI 1.01–1.17, p = 0.022) and albumin-M1 (HR 0.45, 95% CI 0.27–0.74, p = 0.002) were independent prognostic factors for OS. Only neutrophil-M1 was an independent prognostic factor for RFS (HR 1.09, 95% CI 1.02–1.16, p = 0.015).

**Table 2:** Univariable and multivariable analyses for overall and recurrence-free survival.

	Overall survival		Recurrence-free survival	
	Univariable HR	Adjusted HR^†^	Univariable HR	Adjusted HR^††^
Neutrophil preop	1.16 (1.05–1.28) (p = 0.002)		1.14 (1.03–1.25) (p = 0.008)	
Neutrophil-M1	1.09 (1.03–1.16) (p = 0.002)	1.09 (1.01–1.17) (p = 0.022)	1.06 (1.00–1.13) (p = 0.071)	1.09 (1.02–1.16) (p = 0.015)
Lymphocyte preop	0.52 (0.37–0.72) (p <0.001)		0.65 (0.49–0.87) (p = 0.003)	
Lymphocyte-M1	0.59 (0.41–0.84) (p = 0.004)		0.79 (0.58–1.07) (p = 0.126)	
Albumin preop	0.24 (0.17–0.34) (p <0.001)		0.34 (0.24–0.47) (p <0.001)	
Albumin-M1	0.25 (0.18–0.36) (p <0.001)	0.45 (0.27–0.74) (p = 0.002)	0.37 (0.27–0.52) (p <0.001)	

^†^Age, sex, and pathologic stage were added to the model.

^††^Age, sex, body mass index, extent of gastrectomy, and pathologic stage were added to the model.

CI, confidence interval; HR, hazard ratio; M1, 1 month after surgery; preop, preoperative.

Hazard Ratios were presented as HR (95% Confidence Intervals, p value).

**Figure 2:**
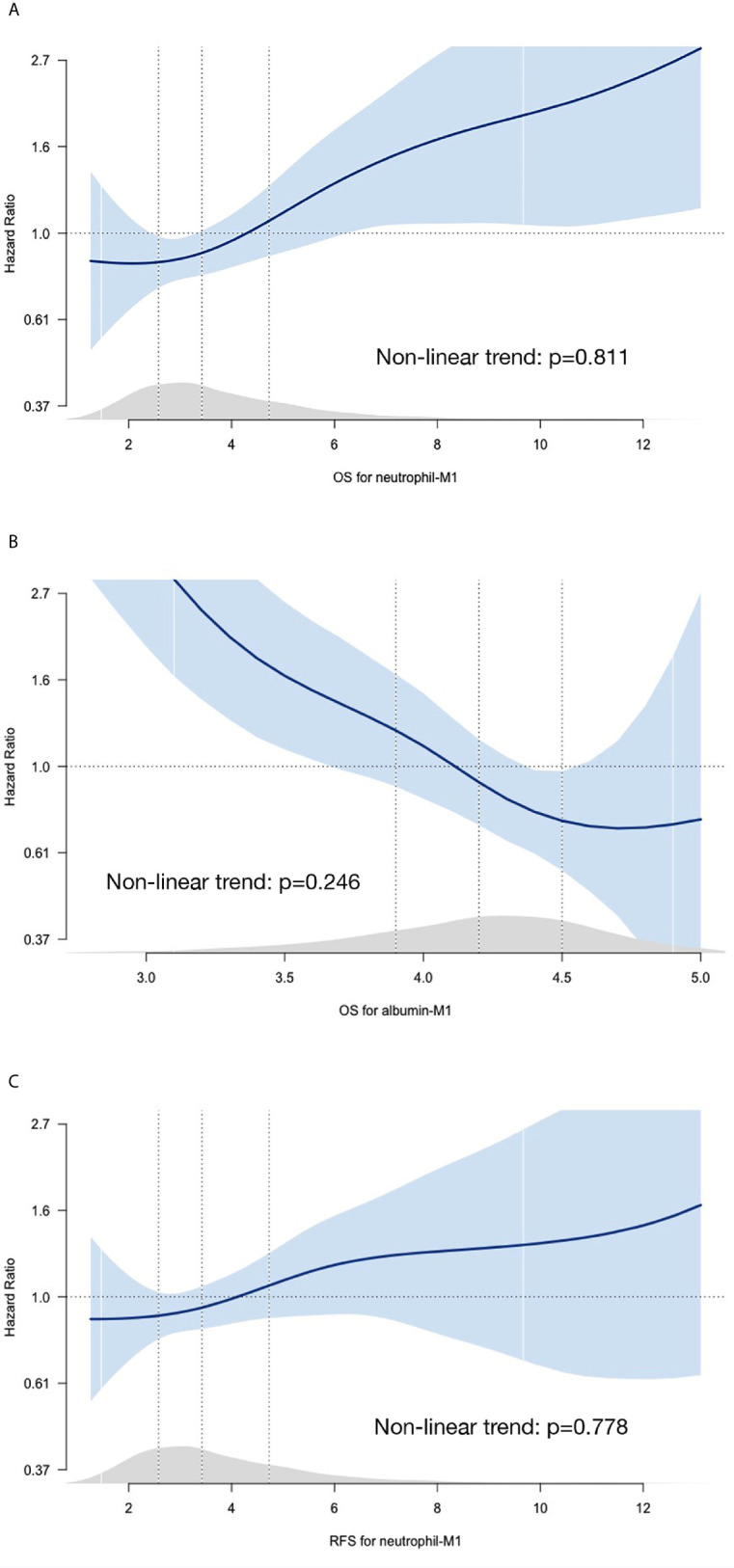
Spline curves for associations between HRs and 1-month absolute values for neutrophil count **(A)**, lymphocyte count **(B)**, and albumin level **(C)**. The blue curves represent penalized smoothing splines for adjusted HRs. Blue-shaded areas represent 95% confidence intervals. HRs were estimated using Cox proportional hazards models adjusted for age, sex, pathologic stage for OS and adjusted for age, sex, pathologic stage, body mass index, and extent of gastrectomy for RFS. Light gray areas just above the x-axis represent density plots. Vertical dotted lines indicate the first quartile, median, and third quartile values. (HR, hazard ratio; M1, 1 month; OS, overall survival; RFS, recurrence-free survival).

Penalized smoothing splines for the associations between 1-month absolute values of independent prognostic parameters and HRs are shown in **Figure 2**. Adjusted spline curves showed that HRs for OS increased as neutrophil-M1 increased and decreased as albumin-M1 increased. HRs for RFS were positively correlated with neutrophil-M1. The overall relationships showed a linear pattern. Conversely, changes from preoperative to 1-month reassessment values (deltas) and HRs exhibited non-linear relationships (**Supplemental Figure 2**); we therefore did not include deltas in further analyses.

### Threshold Selection for Survival Comparisons

We investigated the optimal cut-off values using the maximum log-rank score method and found that a neutrophil-M1 of 4.22 × 10^3^ cell/mm^3^ was the optimal threshold for OS and a neutrophil-M1 of 4.17 × 10^3^ cell/mm^3^ was the optimal threshold for RFS (**Supplemental Figure 3A**). For albumin-M1, 4.1 g/dl was the optimal threshold value for OS (**Supplemental Figure 3B**). For the simplicity of clinical application, 4.2 × 10^3^ cell/mm^3^ and 4.1 g/dl were selected as the thresholds for neutrophil count and albumin level and used to dichotomize patients into groups.

### Comparisons of Overall Survival

**Figure 3:**
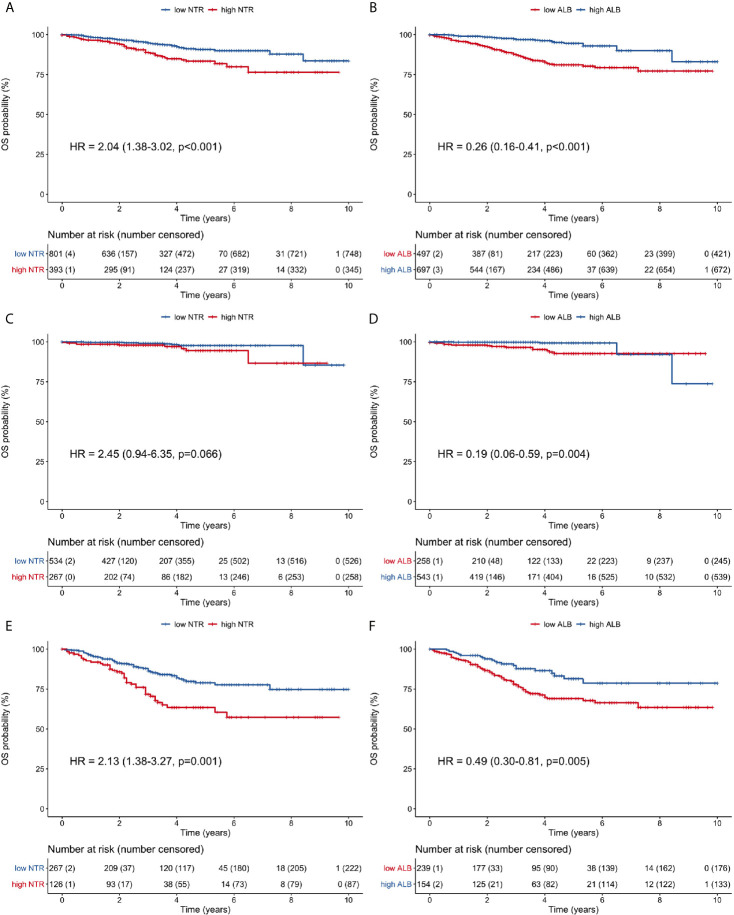
Overall survival curves of patients grouped according to low or high 1-month neutrophil and albumin values. **(A)** all patients, neutrophil count; **(B)** all patients, albumin level; **(C)** stage I patients, neutrophil count; **(D)** stage I patients, albumin level; **(E)** stage II/III patients, neutrophil count; **(F)** stage II/III patients, albumin level. (ALB, albumin; HR, hazard ratio; NTR, neutrophil; OS, overall survival).

Survival curves were compared for all patients (**Figures 3A, B**), stage I patients (**Figures 3C, D**), and stage II/III patients (**Figures 3E, F**). In all patients, high neutrophil-M1 (HR 2.04, 95% CI 1.38–3.02, p <0.001; **Figure 3A**) and low albumin-M1 (HR 0.26, 95% CI 0.16–0.41, p <0.001; **Figure 3B**) were significantly associated with poorer OS. In stage I patients, low albumin-M1 was significantly associated with poorer OS (HR 0.19, 95% CI 0.06–0.59, p = 0.004; **Figure 3D**) but high neutrophil-M1 was not (HR 2.45, 95% CI 0.94–6.35, p = 0.066; **Figure 3C**). For patients with stage II/III cancer, high neutrophil-M1 (HR 2.13, 95% CI 1.38–3.27, p = 0.001; **Figure 3E**) and low albumin-M1 (HR 0.49, 95% CI 0.30–0.81, p = 0.005; **Figure 3F**) were both associated with significantly worse OS.

In stage II/III patients, 1-, 3- and 5-year OS rates were 91.9, 71.7, and 63.5%, respectively, for the high neutrophil-M1 group. By contrast, these rates were 96.2, 86.3, and 78.9% for the low neutrophil-M1 group. Similarly, 1-, 3- and 5-year OS rates were 93.5, 77.6, and 69.1% for the low albumin-M1 group and 96.7, 87.8, and 81.5% for the high albumin-M1 group.

### Overall Survival According to Combined Neutrophil-M1 and Albumin-M1 Values

**Figure 4:**
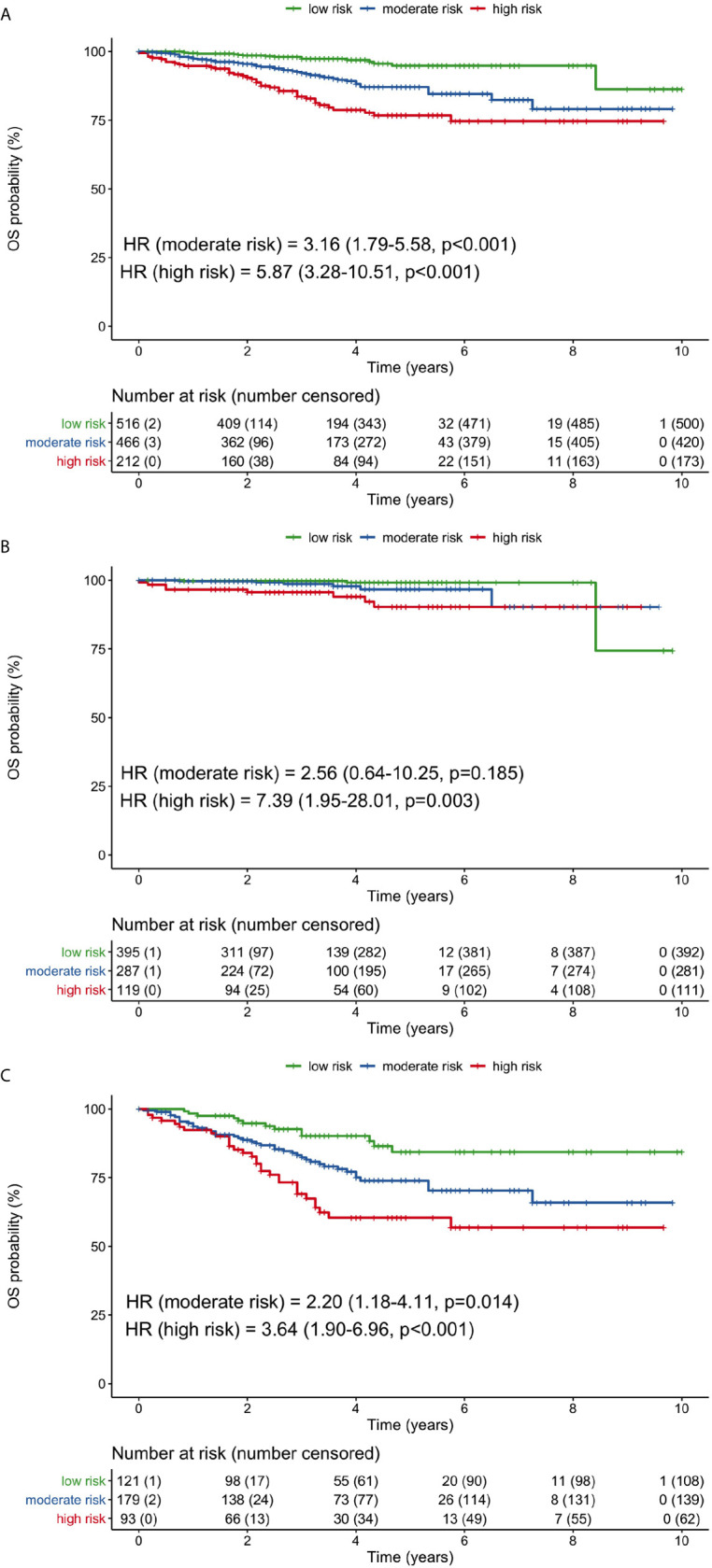
Overall survival curves for low-, moderate-, and high-risk groups based on the combination of 1-month neutrophil and albumin values. **(A)** all patients, **(B)** stage I patients, **(C)** stage II/III patients. (HR, hazard ratio; OS, overall survival).

Neutrophil counts and albumin levels 1 month after surgery were combined for further OS risk stratification. The risk categories were high-risk (high neutrophils and low albumin), moderate-risk (either high neutrophils or low albumin), and low-risk (low neutrophils and high albumin). Compared with the low-risk group, the moderate-risk group (HR 3.16, 95% CI 1.79–45.58, p <0.001) and high-risk group (HR 5.87, 95% CI 3.28–10.51, p <0.001) had significantly worse OS when considering all patients (**Figure 4A**). The 5-year OS rates of the low-, moderate-, and high-risk groups were 94.8, 87.0, and 76.7%, respectively.

In stage I patients, only the high-risk group had significantly poorer OS than the low-risk group (HR 7.39, 95% CI 1.95–28.01, p = 0.003; **Figure 4B**). In stage II/III patients, OS was significantly worse for both the moderate-risk group (HR 2.20, 95% CI 1.18–4.11, p = 0.014) and high-risk group (HR 3.64, 95% CI 1.90–6.96, p <0.001), compared with the low-risk group (**Figure 4C**). The 5-year OS rates in stage II/III patients were 84.3, 73.9, and 60.4% for the low-, moderate, and high-risk groups, respectively.

### Comparisons of Recurrence-Free Survival

**Figure 5:**
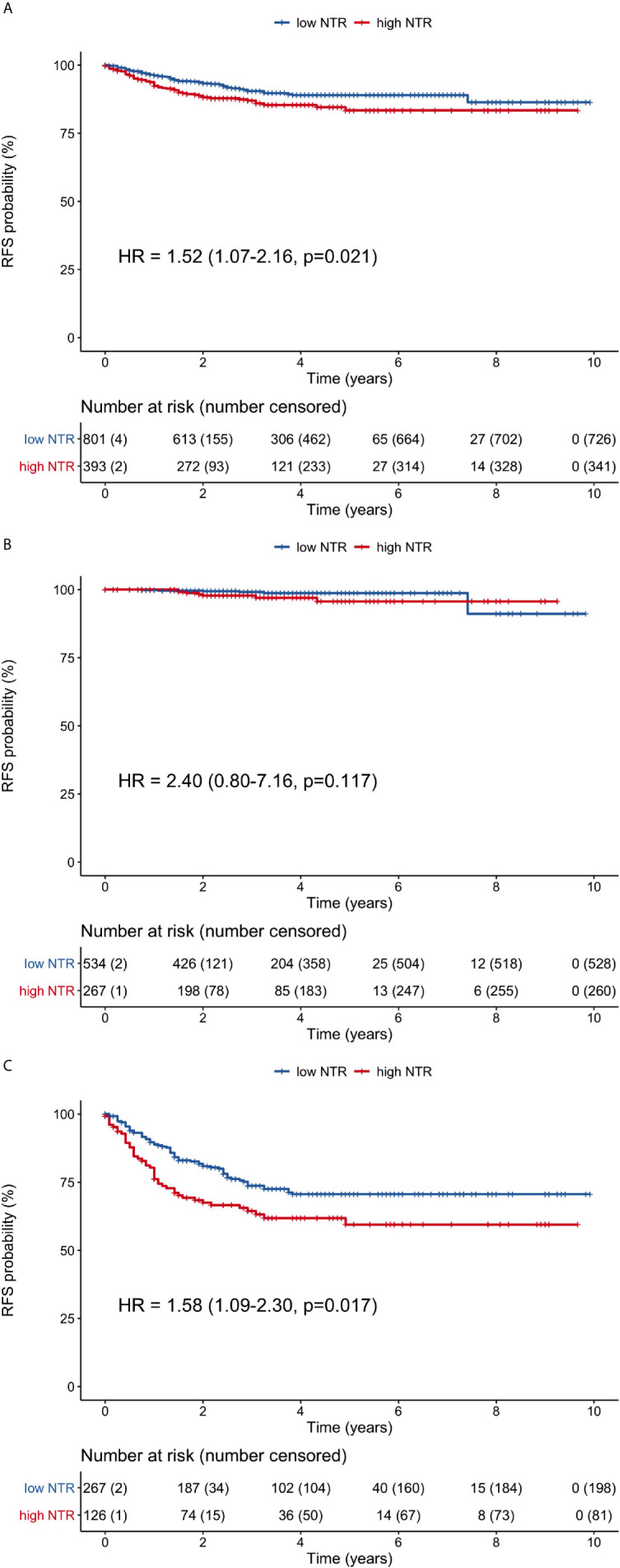
Recurrence-free survival curves of patients grouped according to low or high 1-month neutrophil count. **(A)** all patients, **(B)** stage I patients, **(C)** stage II/III patients. (HR, hazard ratio; NTR, neutrophil; RFS, recurrence-free survival).

The high neutrophil-M1 group had significantly poorer RFS (HR 1.52, 95% CI 1.07–2.16, p = 0.021) when considering all patients (**Figure 5A**). The 1-, 3-, and 5-year RFS rates were 92.4, 87, and 83.4%, respectively, for the high neutrophil-M1 group and 96.2, 90.5, and 89.0% for the low neutrophil-M1 group. RFS did not differ between high and low neutrophil-M1 groups in stage I patients (HR 2.40, 95% CI 0.80–7.16, p = 0.117; **Figure 5B**). However, RFS was significantly worse for the high neutrophil-M1 group in stage II/III patients (HR 1.58, 95% CI 1.09–2.30, p = 0.017; **Figure 5C**). In stage II/III patients, the 1-, 3-, and 5-year RFS rates were 76.1, 64.4, and 59.5%, respectively, for the high neutrophil-M1 group and 88.9, 73.7, and 70.6% for the low neutrophil-M1 group.

## Discussion

The key finding of this study was that specific inflammatory and nutritional parameters measured 1 month after curative surgery for gastric cancer were prognostic factors of long-term outcomes and appeared to be more useful than preoperative values. In univariable analysis, several preoperative and postoperative values were significantly associated with long-term prognosis. However, in multivariable analysis adjusted for clinical parameters, 1-month postoperative neutrophil count and albumin level were identified as independent prognostic factors of OS, and 1-month postoperative neutrophil count was also independent prognostic factors of RFS. When we divided patients into different risk groups using threshold values, combining neutrophil count and albumin level increased their prognostic ability for OS. Patients in the high-risk group (high neutrophil count and low albumin level) had poorer OS. For recurrence-free survival (RFS), patients in the high-risk group based on a high neutrophil count showed higher recurrence rate. Although the prognostic ability of these parameters was demonstrated when considering all patients included in the study, their prognostic value was particularly obvious in patients with stage II/III cancer.

One of the most important findings of this study is the relationship between the neutrophil count measured 1 month after surgery and both OS and RFS. There is little previous evidence regarding the prognostic value of neutrophil counts, as most studies have not evaluated neutrophil count as a single independent factor. Its prognostic value has primarily been evaluated using neutrophil-containing parameters, such as NLR, SII, or neutrophil–albumin ratio (NAR) (3, 13, 14). Our previous study demonstrated that preoperative neutrophil count was a strong independent prognostic factor for OS and RFS after adjusting for clinical and pathologic characteristics. In the present study, which contained a larger number of patients and extensive updates regarding chemotherapy and survival details, we found that postoperative neutrophil counts may be more useful than preoperative counts. The association between postoperative neutrophil count and both OS and RFS may be explained by several possible mechanisms. Various neutrophil-related elements (such as tumor-associated neutrophils, vascular endothelial growth factor, and neutrophil extracellular traps) may promote mutations leading to tumorigenesis and tumor cell proliferation, contribute to tumor-related angiogenesis, induce cancer cells to metastasize, inhibit T-cell activity against tumor cells, and increase adhesion between circulating tumor cells and organs (15–19). All of these mechanisms suggest that high neutrophil counts may lead to a poorer prognosis. Conversely, it is possible that elevated neutrophil counts are simply the result of ongoing tumor-associated inflammation and do not directly contribute to a worse prognosis.

Albumin measured 1 month after surgery was also identified as a prognostic indicator for OS. Albumin is well-established marker of nutritional status. With increasing evidence of the relationship between hypoalbuminemia and poor prognosis, the prognostic value of a low albumin level has been tested and validated in several composite ratios or cumulative scores for a variety of malignancies (20–22). In this study, we defined three risk groups based on neutrophil count and albumin values to assess the clinical applicability of our findings. Using this stratification system, which was similar to the categorization strategy used for the albumin-neutrophil combined prognostic grade in patients with lung cancer (23), we observed a significantly worse prognosis in the high-risk group than in the low-risk group. In our cohort, 5-year OS rate was 94.8% for the low-risk group but only 76.7% for the high-risk group. This survival difference between risk groups was most apparent in patients with stage II/III cancer: 84.3% for the low-risk group and 60.4% for the high-risk group.

The literature exploring the prognostic value of postoperative reassessments is limited. For patients with gastric cancer, low PNI in the postoperative period has been associated with worse survival, regardless of preoperative values (4, 5). This finding is consistent with our results. In a study evaluating NLR before and after surgery, high NLR values in both periods were associated with poorer survival (3). Although dynamic changes such as delta values between preoperative and postoperative values have been evaluated for various organ cancers, postoperative absolute values have been most commonly evaluated for colorectal cancer, hepatocellular carcinoma, and pancreatic carcinoma (24–28). Most previous research examined NLR and PNI, although postoperative changes for PLR and SII have also been evaluated; all of these parameters were associated with prognosis (29, 30). However, these parameters were complex scores combining two or more parameters. By contrast, we have focused primarily on simple parameters, such as neutrophil count, lymphocyte count, and albumin level. As noted in our previous study, simple parameters were found to adequately reflect inflammatory status. We also evaluated various complex parameters, including NLR, PLR, and PNI, but they were not superior to the simple parameters (data not shown).

We used absolute values in our analysis instead of delta values primarily because non-linear relationships were observed between delta values and HRs. Non-linear relationships would lead to inaccuracies if delta values were categorized as decreased or increased by dichotomizing the values using single cut-off values. To overcome this problem, patients could be divided into multiple subgroups based on delta values; however, multiple subgroups would make risk assessment more complicated and less useful for clinical practice. In the present study, we used only thresholds for absolute values with linear HR relationships. The relevant thresholds used in this study were calculated statistically and assessed clinically. These thresholds require validation in further clinical studies.

One month after curative surgery can be considered an ideal time for reassessment. It is late enough for the inflammatory response to surgical intervention to resolve and early enough to allow appropriate decision making before initiating adjuvant therapy. In the present study, we excluded patients with major perioperative complications to avoid potential bias associated with inflammation secondary to these complications. Previous studies of patients with gastric cancer have evaluated 3- or 6-month postoperative values as potential prognostic factors; however, the interpretation of the results at these times may be unsuitable because laboratory data were complicated by the use of adjuvant chemotherapy (4, 5, 26, 30).

Despite robust methodology and use of a comprehensive database, the present study had some limitations. First, all patients underwent surgery in a single tertiary center, and all operations were performed by the same surgeon. Experience bias may limit the generalizability of our findings. Second, because of the retrospective study design, it was not possible to use immune parameters as a diagnostic tool during long-term follow-up. However, by demonstrating the link between survival and post-treatment laboratory values, our results may form the basis for future investigations of the relationship between recurrence and laboratory values. Third, although all patients were treated according to current guidelines, adjuvant treatment may have varied and impacted the outcomes.

In conclusion, reassessment of a patient’s inflammatory and nutritional status 1 month after curative surgery may provide important information regarding long-term prognosis and may be more useful than preoperative values. These parameters can be used to stratify patients within the same pathologic stage into different prognostic groups.

## Data Availability Statement

The raw data supporting the conclusions of this article will be made available by the authors, without undue reservation.

## Ethics Statement

The studies involving human participants were reviewed and approved by Institutional Review Board of Severance Hospital. Written informed consent for participation was not required for this study in accordance with the national legislation and the institutional requirements.

## Author Contributions

Authors made substantial contributions to conception and design, and/or acquisition of data, and/or analysis and interpretation of data; participated in drafting the article or revising it critically for important intellectual content; and gave final approval of the version to be published. Besides, AG, MC, and H-IK designed the study and prepared study protocol. Y-MK, J-HC, WH, and H-IK contributed to data acquisition. AG, Y-MK, and H-IK contributed to data analysis including statistical analysis. AG, J-HC, WH, and H-IK contributed to interpretation of article. AG, MC, WH, and H-IK contributed to writing of article. J-HC, WH, and H-IK critically revised the manuscript for important intellectual content. All authors contributed to the article and approved the submitted version.

## Funding

This work was supported by the National Research Foundation of Korea (NRF) grant funded by the Korea government (MSIT) (no. 2016R1A2B4014984 and no. 2019R1H1A2079953).

## Conflict of Interest

The authors declare that the research was conducted in the absence of any commercial or financial relationships that could be construed as a potential conflict of interest.

## Supplementary Material

Supplemental Figure 1 Correlation matrix among the preoperative and 1-month neutrophil count, lymphocyte count and albumin values. Positive correlations are displayed in blue and negative correlations in red color. Color intensity and the circle size are proportional to the correlation coefficients. Numbers also show the correlation coefficients between parameters. (preop, preoperative, M1, 1 month).

Supplemental Figure 2 Spline curves for associations between HRs and delta values (1-month minus preoperative) for neutrophil count **(A)**, lymphocyte count **(B)**, and albumin level **(C)**. The blue curves represent penalized smoothing splines for adjusted HRs. Blue-shaded areas represent 95% confidence intervals. HRs were estimated using Cox proportional hazards models adjusted for age, sex, and pathologic stage for OS and adjusted for age, sex, pathologic stage, body mass index, and extent of gastrectomy for RFS. Light gray areas just above the x-axis represent density plots. Vertical dotted lines indicate the first quartile, median, and third quartile values. (HR, hazard ratio; M1, 1 month; OS, overall survival; RFS, recurrence-free survival).

Supplemental Figure 3 Various cut-off values of neutrophil count **(A)** and albumin level **(B)** and their associated log-rank test scores for survival. (OS, overall survival; RFS, recurrence-free survival).

## References

[1] Cheong JH Yang HK Kim H Kim WH Kim YW Kook MC. Predictive test for chemotherapy response in resectable gastric cancer: a multi-cohort, retrospective analysis. Lancet Oncol (2018) 19(5):629–38. 10.1016/S1470-2045(18)30108-6 29567071

[2] Guner A Kim HI. Biomarkers for Evaluating the Inflammation Status in Patients with Cancer. J Gastric Cancer (2019) 19(3):254–77. 10.5230/jgc.2019.19.e29 PMC6769371 31598370

[3] Szor DJ Dias AR Pereira MA Ramos M Zilberstein B Cecconello I. Neutrophil-lymphocyte ratio change after curative gastrectomy for gastric cancer: a subgroup analysis. Einstein (Sao Paulo) (2020) 18:eAO4860. 10.31744/einstein_journal/2020AO4860 31778466 PMC6896601

[4] Park SH Lee S Song JH Choi S Cho M Kwon IG. Prognostic significance of body mass index and prognostic nutritional index in stage II/III gastric cancer. Eur J Surg Oncol (2020) 46:620–5. 10.1016/j.ejso.2019.10.024 31668977

[5] Oh SE Choi MG Seo JM An JY Lee JH Sohn TS. Prognostic significance of perioperative nutritional parameters in patients with gastric cancer. Clin Nutr (2019) 38(2):870–6. 10.1016/j.clnu.2018.02.015 29503057

[6] Migita K Matsumoto S Wakatsuki K Ito M Kunishige T Nakade H. A decrease in the prognostic nutritional index is associated with a worse long-term outcome in gastric cancer patients undergoing neoadjuvant chemotherapy. Surg Today 47(8):1018–26. 10.1007/s00595-017-1469-y 28251372

[7] Guner A Kim SY Yu JE Min IK Roh YH Roh C. Parameters for Predicting Surgical Outcomes for Gastric Cancer Patients: Simple Is Better Than Complex. Ann Surg Oncol (2018) 25(11):3239–47. 10.1245/s10434-018-6684-2 30069658

[8] Guner A Kim HI. ASO Author Reflections: Parameters for Predicting Surgical Outcomes for Gastric Cancer Patients: Simple Is Better Than Complex. Ann Surg Oncol (2018) 25(Suppl 3):699–700. 10.1245/s10434-018-6725-x 30167908

[9] D. W. G. Guideline Committee of the Korean Gastric Cancer Association and P. Review. Korean Practice Guideline for Gastric Cancer 2018: an Evidence-based, Multi-disciplinary Approach. J Gastric Cancer (2019) 19(1):1–48. 10.5230/jgc.2019.19.e8 30944757 PMC6441770

[10] A. Japanese Gastric Cancer. Japanese gastric cancer treatment guidelines 2014 (ver. 4). Gastric Cancer (2017) 20(1):1–19. 10.1007/s10120-016-0622-4 PMC5215069 27342689

[11] Amin M Edge S Greene F Byrd D Brookland R Washington M. AJCC Cancer Staging Manual. 8th edition. American Joint Commission on Cancer. New York: Springer International Publishing (2017).

[12] Hosmer D Lemeshow S May S. Applied Survival Analysis: Regression Modeling of Time to Event Data. Hoboken: Wiley, New Jersey (2008).

[13] Okugawa Y Toiyama Y Yamamoto A Omura Y Kusunoki K Yin C. Modified neutrophil-platelet score as a promising marker for stratified surgical and oncological outcomes of patients with gastric cancer. Surg Today (2020) 50(3):223–31. 10.1007/s00595-019-01873-y 31485750

[14] Dong M Shi Y Yang J Zhou Q Lian Y Wang D. Prognostic and clinicopathological significance of systemic immune-inflammation index in colorectal cancer: a meta-analysis. Ther Adv Med Oncol (2020) 12:1758835920937425. 10.1177/1758835920937425 32699557 PMC7357045

[15] Gaudry M Bregerie O Andrieu V El Benna J Pocidalo MA Hakim J. Intracellular pool of vascular endothelial growth factor in human neutrophils. Blood (1997) 90(10):4153–61. 9354686

[16] Hormbrey E Han C Roberts A McGrouther DA Harris AL. The relationship of human wound vascular endothelial growth factor (VEGF) after breast cancer surgery to circulating VEGF and angiogenesis. Clin Cancer Res (2003) 9(12):4332–9. 14555503

[17] Tazzyman S Lewis CE Murdoch C. Neutrophils: key mediators of tumour angiogenesis. Int J Exp Pathol (2009) 90(3):222–31. 10.1111/j.1365-2613.2009.00641.x PMC2697547 19563607

[18] Shaul ME Fridlender ZG. Tumour-associated neutrophils in patients with cancer. Nat Rev Clin Oncol (2019) 16(10):601–20. 10.1038/s41571-019-0222-4 31160735

[19] Wang Y Zhai J Zhang T Han S Zhang Y Yao X. Tumor-Associated Neutrophils Can Predict Lymph Node Metastasis in Early Gastric Cancer. Front Oncol (2020) 10:570113:570113. 10.3389/fonc.2020.570113 33072602 PMC7537418

[20] Dolan RD McSorley ST Park JH Watt DG Roxburgh CS Horgan PG. The prognostic value of systemic inflammation in patients undergoing surgery for colon cancer: comparison of composite ratios and cumulative scores. Br J Cancer 119(1):40–51. 10.1038/s41416-018-0095-9 PMC6035216 29789606

[21] Tingle SJ Severs GR Goodfellow M Moir JA White SA. NARCA: A novel prognostic scoring system using neutrophil-albumin ratio and Ca19-9 to predict overall survival in palliative pancreatic cancer. J Surg Oncol 118(4):680–6. 10.1002/jso.25209 30196571

[22] Tawfik B Mokdad AA Patel PM Li HC Huerta S. The neutrophil to albumin ratio as a predictor of pathological complete response in rectal cancer patients following neoadjuvant chemoradiation. Anticancer Drugs 27(9):879–83. 10.1097/CAD.0000000000000411 27434664

[23] Sun H Hu P Shen H Dong W Zhang T Liu Q. Albumin and Neutrophil Combined Prognostic Grade as a New Prognostic Factor in Non-Small Cell Lung Cancer: Results from a Large Consecutive Cohort. PloS One 10(12):e0144663. 10.1371/journal.pone.0144663 PMC4682770 26656866

[24] Guo D Han A Jing W Chen D Jin F Li M. Preoperative to postoperative change in neutrophil-to-lymphocyte ratio predict survival in colorectal cancer patients. Future Oncol 14(12):1187–96. 10.2217/fon-2017-0659 29302993

[25] Li Z Zhao R Cui Y Zhou Y Wu X. The dynamic change of neutrophil to lymphocyte ratio can predict clinical outcome in stage I-III colon cancer. Sci Rep 8(1):9453. 10.1038/s41598-018-27896-y PMC6013456 29930287

[26] Ikeguchi M Goto K Watanabe J Urushibara S Osaki T Endo K. Clinical importance of preoperative and postoperative prognostic nutritional index in patients with pancreatic ductal adenocarcinoma. Ann Hepatobiliary Pancreat Surg 23(4):372–6. 10.14701/ahbps.2019.23.4.372 PMC6893056 31825004

[27] Lee YJ Kim WR Han J Han YD Cho MS Hur H. Prognostic Impact of Immunonutritional Status Changes During Preoperative Chemoradiation in Patients With Rectal Cancer. Ann Coloproctol 32(6):208–14. 10.3393/ac.2016.32.6.208 PMC5256249 28119863

[28] Zhang X Li C Wen T Peng W Yan L Yang J. Postoperative Prognostic Nutritional Index Predicts Survival of Patients with Hepatocellular Carcinoma within Milan Criteria and Hypersplenism. J Gastrointest Surg 21(10):1626–34. 10.1007/s11605-017-3414-1 28523484

[29] Peng W Li C Zhu WJ Wen TF Yan LN Li B. Prognostic value of the platelet to lymphocyte ratio change in liver cancer. J Surg Res (2015) 194(2):464–70. 10.1016/j.jss.2014.12.021 25577142

[30] Wang BL Tian L Gao XH Ma XL Wu J Zhang CY. Dynamic change of the systemic immune inflammation index predicts the prognosis of patients with hepatocellular carcinoma after curative resection. Clin Chem Lab Med (2016) 54:1963–9. 10.1515/cclm-2015-1191 27010778

