# Supplementary figures and images for: Prognostic Value of Postoperative Neutrophil and Albumin: Reassessment One Month After Gastric Cancer Surgery
Source: Front Oncol. 2021 Mar 23;11:633924. doi: 10.3389/fonc.2021.633924 (PMC8023044; doi:10.3389/fonc.2021.633924)

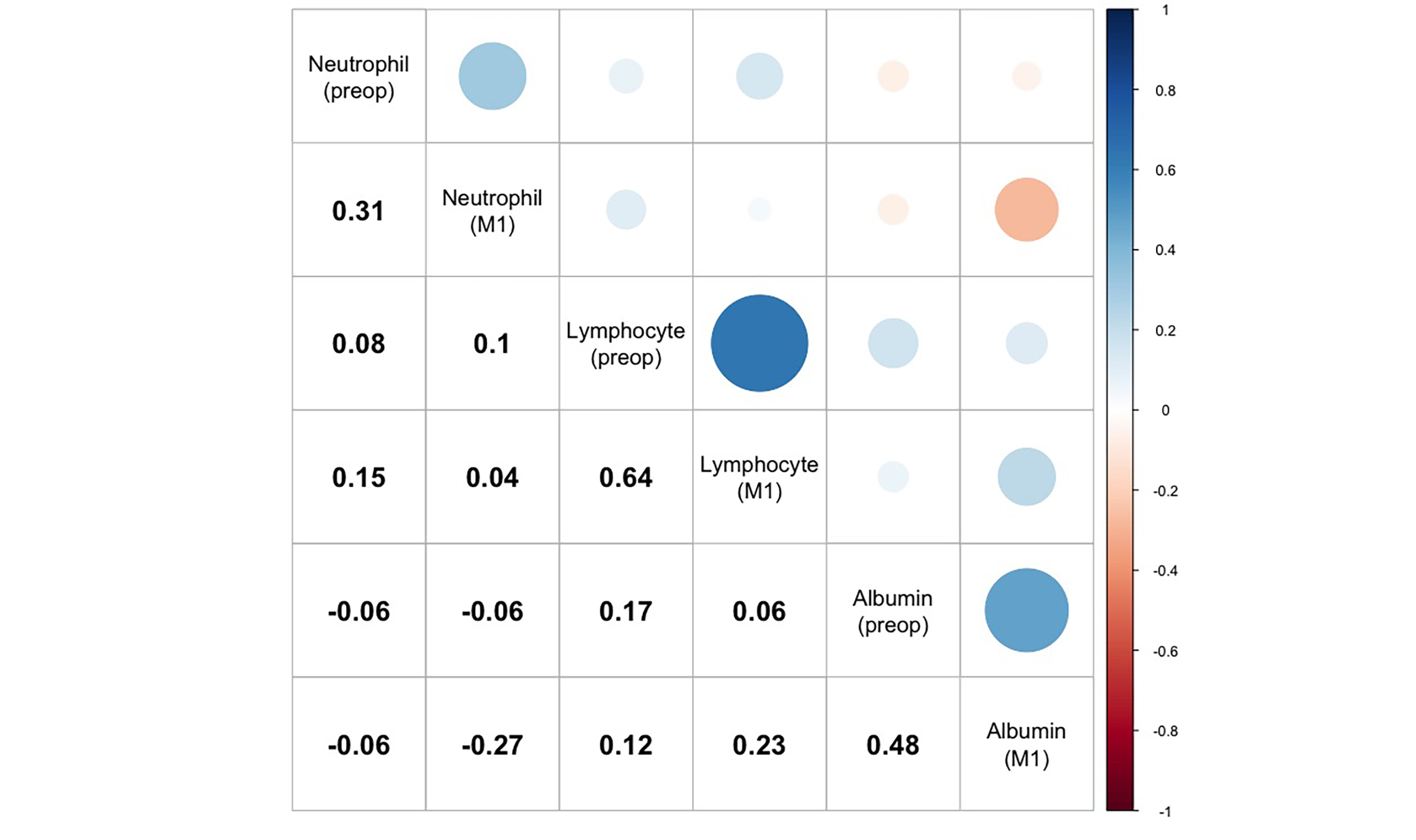

Supplement: Supplemental Figure 1 — Correlation matrix among the preoperative and 1-month neutrophil count, lymphocyte count and albumin values. Positive correlations are displayed in blue and negative correlations in red color. Color intensity and the circle size are proportional to the correlation coefficients. Numbers also show the correlation coefficients between parameters. (preop, preoperative, M1, 1 month). [file Image_1.jpeg]

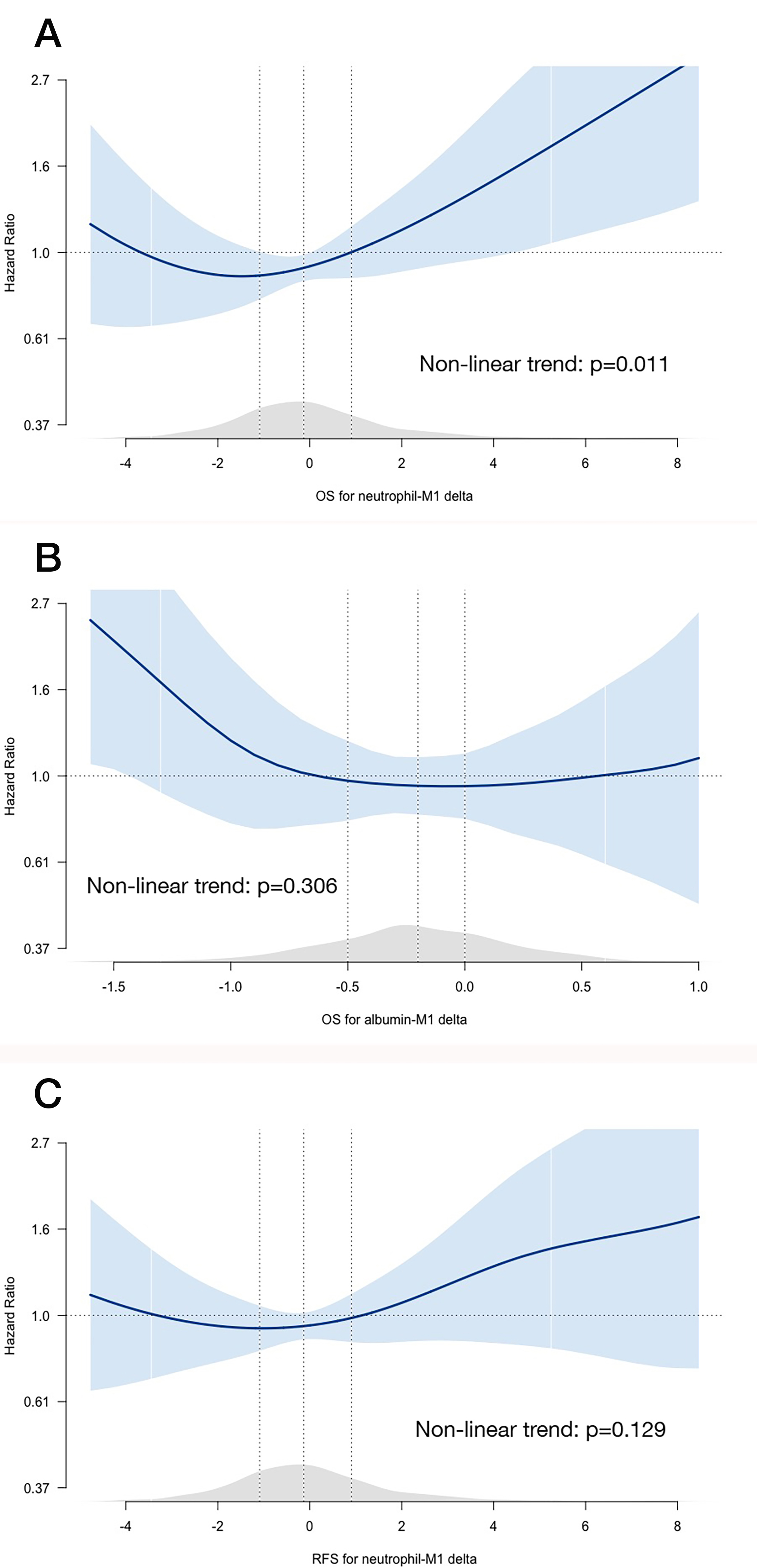

Supplement: Supplemental Figure 2 — Spline curves for associations between HRs and delta values (1-month minus preoperative) for neutrophil count (A), lymphocyte count (B), and albumin level (C). The blue curves represent penalized smoothing splines for adjusted HRs. Blue-shaded areas represent 95% confidence intervals. HRs were estimated using Cox proportional hazards models adjusted for age, sex, and pathologic stage for OS and adjusted for age, sex, pathologic stage, body mass index, and extent of gastrectomy for RFS. Light gray areas just above the x-axis represent density plots. Vertical dotted lines indicate the first quartile, median, and third quartile values. (HR, hazard ratio; M1, 1 month; OS, overall survival; RFS, recurrence-free survival). [file Image_2.jpeg]

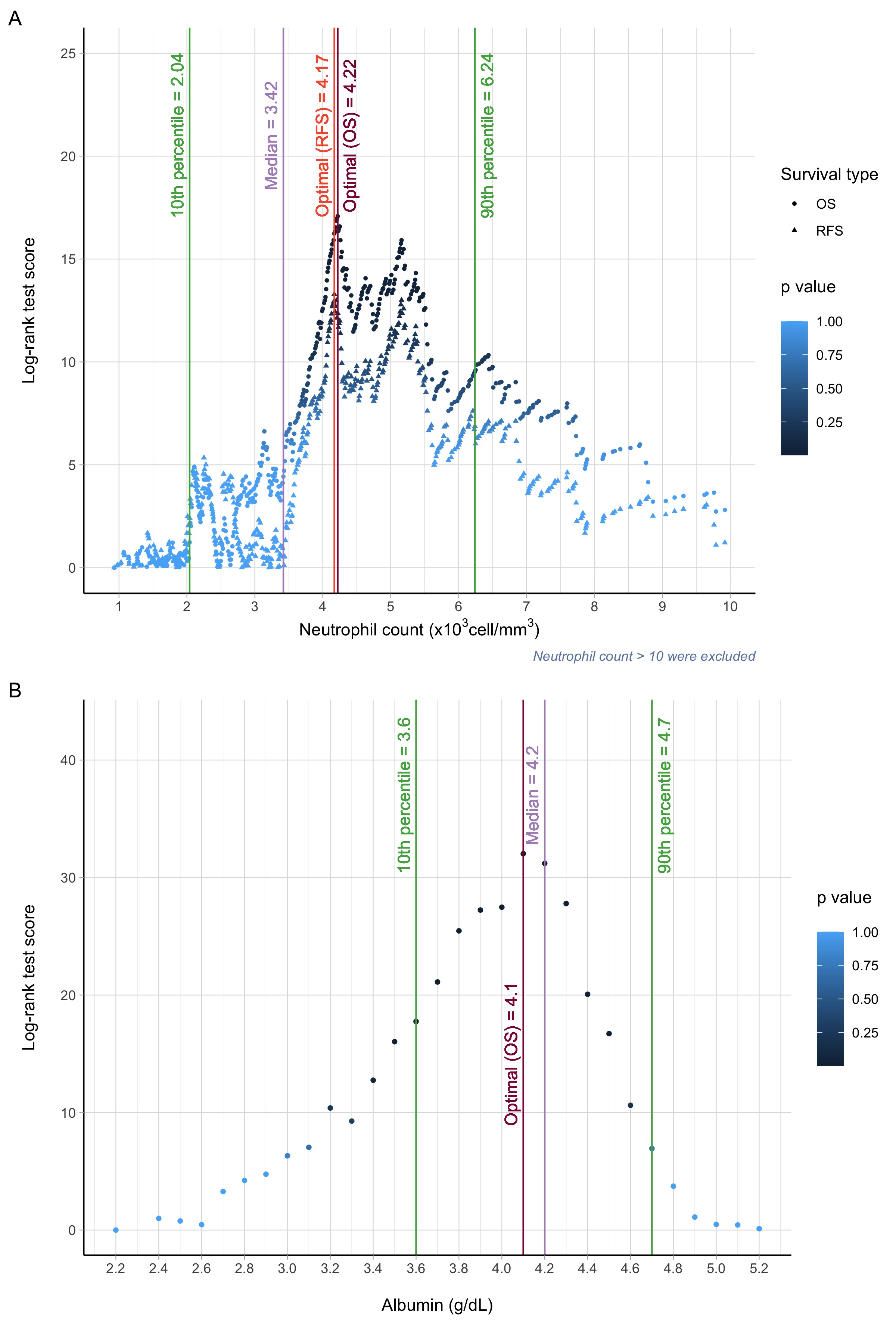

Supplement: Supplemental Figure 3 — Various cut-off values of neutrophil count (A) and albumin level (B) and their associated log-rank test scores for survival. (OS, overall survival; RFS, recurrence-free survival). [file Image_3.jpeg]
